# Dissecting the mechanism of temozolomide resistance and its association with the regulatory roles of intracellular reactive oxygen species in glioblastoma

**DOI:** 10.1186/s12929-021-00717-7

**Published:** 2021-03-08

**Authors:** Chia-Hung Chien, Wei-Ting Hsueh, Jian-Ying Chuang, Kwang-Yu Chang

**Affiliations:** 1 National Institute of Cancer Research, National Health Research Institutes, 367 Sheng-Li Road, Tainan, 70456 Taiwan; 2 Department of Oncology, College of Medicine, National Cheng Kung University Hospital, National Cheng Kung University, Tainan, Taiwan; 3 Center for Neurotrauma and Neuroregeneration, Taipei Medical University, Taipei, Taiwan; 4 The Ph.D. Program for Neural Regenerative Medicine, Taipei Medical University, Taipei, Taiwan

**Keywords:** Temozolomide resistance, DNA damage, Glioma stem‐like cells, Reactive oxygen species

## Abstract

Glioblastoma is the most common primary malignant brain tumor that is usually considered fatal even with treatment. This is often a result for tumor to develop resistance. Regarding the standard chemotherapy, the alkylating agent temozolomide is effective in disease control but the recurrence will still occur eventually. The mechanism of the resistance is various, and differs in terms of innate or acquired. To date, aberrations in O^6^-methylguanine-DNA methyltransferase are the clear factor that determines drug susceptibility. Alterations of the other DNA damage repair genes such as DNA mismatch repair genes are also known to affect the drug effect. Together these genes have roles in the innate resistance, but are not sufficient for explaining the mechanism leading to acquired resistance. Recent identification of specific cellular subsets with features of stem-like cells may have role in this process. The glioma stem-like cells are known for its superior ability in withstanding the drug-induced cytotoxicity, and giving the chance to repopulate the tumor. The mechanism is complicated to administrate cellular protection, such as the enhancing ability against reactive oxygen species and altering energy metabolism, the important steps to survive. In this review, we discuss the possible mechanism for these specific cellular subsets to evade cancer treatment, and the possible impact to the following treatment courses. In addition, we also discuss the possibility that can overcome this obstacle.

## Background

Glioblastoma (glioblastoma multiforme, GBM) is the most common primary malignant brain tumor. In the United States, the annual incidence is 5.26 per 100,000 population or 17,000 new diagnoses per year [1]. GBM is the highest grade of glioma by histologic definition, and is the most common and the most aggressive type among them [2]. In the latest version of World Health Organization classification, GBM is categorized based on presence or absence of isocitrate dehydrogenase (IDH) mutation [3]. The former usually appears as secondary tumor of the lower grade diseases, and occurs in about the forth to fifth decades of ages. The latter accounts for 90 % of the cases, with most of them occurring in the sixth to seventh decades of ages. A recent study with The Cancer Genome Atlas (TCGA) project had further identified four distinct subgroups for advanced glioma based on the molecular difference: proneural, neural, classical, and mesenchymal [4]. The subclassification differed in genetic expression and the factors to determine the survival advantages [5]. For example, IDH-mutation disease had relatively longer duration of the disease course [3], and thus, recognition of the proneural type that consisted more of IDH1/2 mutation had its clinical significance [4, 6, 7]. The aberrations of genes in neural subgroup were more typified of neuron markers [4]. Finally, the classical and the mesenchymal types, which were more related to EGFR and NF1 aberrations, respectively, benefit with more intensive treatment. Altogether, identifying the subgroup characteristics would potentially support clinicians in making the treatment decision [4].

Comparing to the other malignancies, GBM is relatively rare but desperate. The 2-year survival rate is only 26.5 %, which has one of the worst outcomes regarding the advancement of latest treatment strategies [8]. Even applying the standard management with surgical intervention is sometimes questionable to gain benefit in disease control. In general, extensive resection is suggested to yield survival advantage, and the relatively conservative stereotactic biopsy is performed only in patients who have inoperable tumors that are located in critical areas [8]. This procedure, however, often accompanies with neurological complications, limiting its extent for tumor eradication. As thus, aggressive management with adjuvant therapy is necessary to maximize the treatment effect. Disappointedly, only limited reagents are considered contributable to disease control. The most widely used anti-tumor agent is radiotherapy and temozolomide (TMZ), a chemotherapy that acts as an alkylating agent to cause lethal DNA damage. The other drugs such as carmustine (BCNU) sponge, alternating electric field therapy (tumor-treating fields device, or TTFields), bevacizumab, cisplatin are active but again, with modest effect in disease control. Novel targeting therapies, such as peptide cancer vaccine against EGFR variant III or immune checkpoint inhibitors, were expected to be successful but ended up with disappointment [9, 10]. In summary, not much option is available for treatment.

As being the standard systemic treatment agent, TMZ is a second-generation imidazotetrazine lipophilic prodrug. Currently, it is perhaps the most important systemic drug in GBM treatment. It works by hydrolyzing into its active metabolite 5-(3-dimethyl-1-triazenyl) imidazole-4-carboxamide. The reactive methyldiazonium ion is then formed to methylation-associated residues in the DNA molecule at O^6^- and N^7^-methylguanine (MeG) or N^7^-methyladenine (MeA). Regarding O^6^-MeG, when DNA mismatch repair (MMR) enzymes attempt to excise the modified nucleotide, they generate single- and double-strand breaks in the DNA that lead to activation of apoptotic pathways if no further repairment is available [11]. The drug has been proven with robust data alone or with radiotherapy in clinical trials and retrospective studies, earning the unequivocal role for treatment of the disease [11–14]. In a clinical trial, patients received standard TMZ/radiotherapy yielded significantly better survival, with 9.8 % of them survived five years after diagnosis [12]. In the TMZ era, the mean survival of glioblastoma in patients age 20–29 could be as long as 31.9 months, highlighting the significant effect of the drug [13]. Those with extremely long survival of more than 4 years are featured with lacking O^6^-methylguanine-DNA methyltransferase (MGMT, or O^6^-alkylguanine DNA alkyltransferase) but not the other molecular subclassification [15]. Most of all, the drug is capable of penetrating the blood brain barrier, giving the area under curve of cerebrospinal fluid approximately 20 % of the systemic TMZ exposure [16]. With its superb activity in GBM, the drug has been approved for the treatment with radiation and after for maintenance.

Even with the successful data after introduction of TMZ, the disease, however, remains far from optimal control in clinical aspect. Limited therapeutic efficacy has been a major issue due to eventual failure of the treatment. Despite of the initial response, development of resistance is almost inevitable, with 90 % of patients suffering from early disease recurrence [12]. The remaining course after recurrence is often dismal, and exhibits more deteriorated and resistant nature to the early one. In this article, we review the probable causes leading to the failure of this chemotherapeutic agent. This includes the theories from DNA to cellular levels, and thus, providing an overall understanding of the resistant mechanism against TMZ.

## Limitation in theory of TMZ resistance related to DNA repairing mechanism

**Fig. 1:**
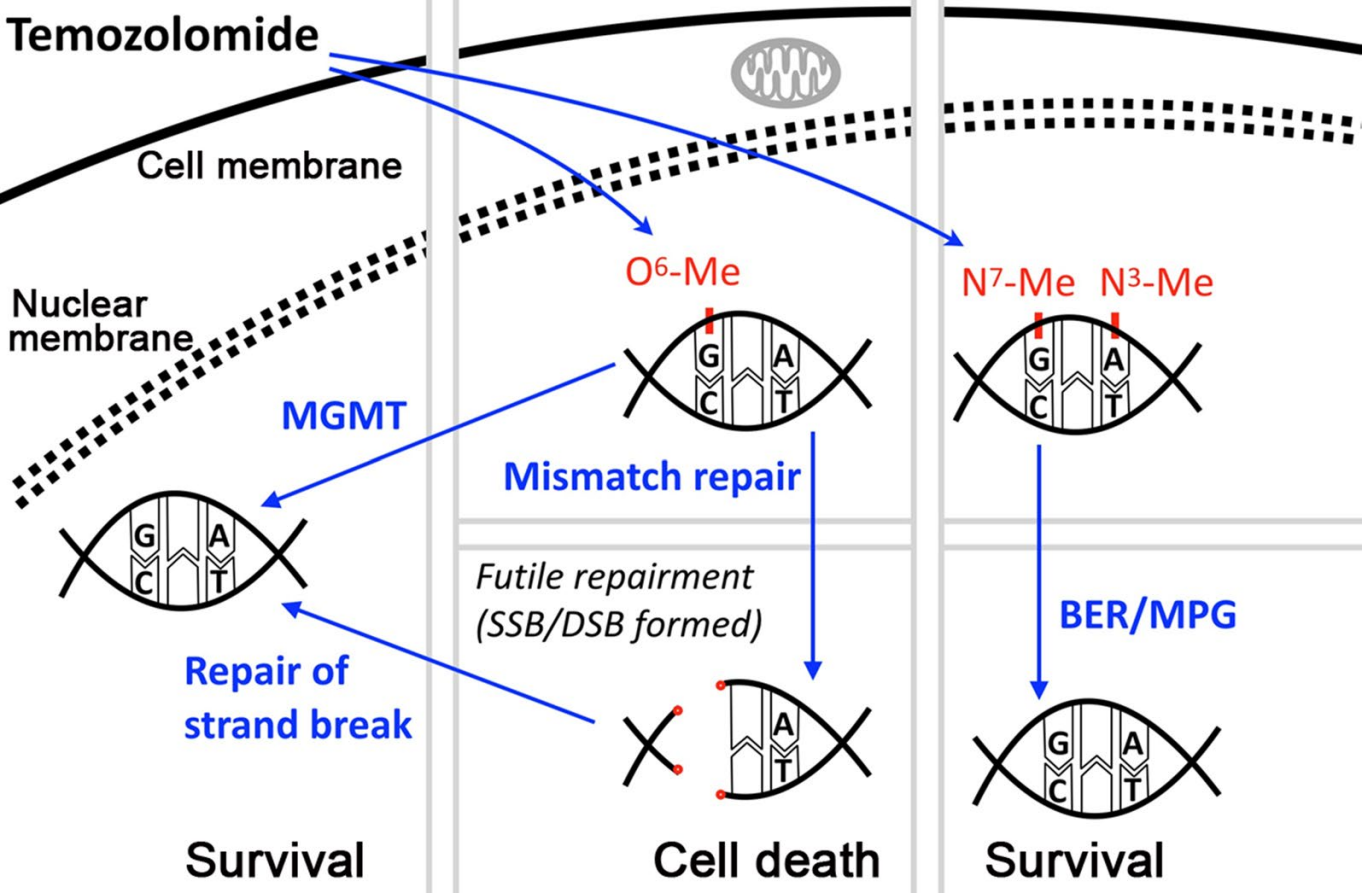
Schema of classical model of the pharmacology mechanism causing temozolomide (TMZ) resistance. Methylation of O^6^, N^3^, and N^7^ in DNA can be modulated by the drug. Repairing response through DDR can cause divergent results. As the notable factor targeting majorly on O^6^-methylguanine, presence of MGMT leads to salvage for the cells to survive, and thus, will have negative impact to drug susceptibility in terms of the drug effect. Without adequate rescuing action, futile repairment will lead to single- or double-strand break, leading to associated reaction causing cell death. *Me* methylation, *SSB* single-strand break, *DSB* double-strand break, *BER* base excision repair, *MPG* N-methylpurine DNA glycosylase

Understanding the resistance mechanism is necessary to help to develop potential strategies against the dilemma. To date, MGMT is the best-known factor leading to resistance of TMZ in GBM [17, 18] (Fig. 1). Inarguably, the factor has been clinically evidenced to contribute the innate tumor resistance against TMZ but not radiotherapy [19]. Presence of MGMT enables cells to remove the alkyl groups from the O^6^ position of guanine to reverse the cytotoxicity of TMZ [20]. The challenge for this factor to predict treatment outcome, however, is to determine the most reliable and relevant detecting methods. Unfortunately to date, this remained unconfirmed [21]. Because of complicated regulatory mechanism, relying upon promoter research may not be sufficient [22]. This would leave small but certain number of patients to benefit from chemoradiotherapy even with lacking MGMT promoter methylation [23]. In addition, presence of this DNA repairing gene provides only partial explanation for the resistance because about half of patients express the protein and 43–47.5 % of patients have MGMT promoter methylation silenced [24, 25]. Nevertheless, our understanding of MGMT tends to be helpful in predicting the prognosis of disease. In a large phase III clinical trials with 833 newly diagnosed patients receiving standard or dose-dense TMZ, stratification with the status of the MGMT promoter identified improved survival data in methylated groups [26]. Moreover, the role for the gene status to guide application of TMZ is in suggestive. Two phase III trials, one in general and the other in elderly populations, suggested effectiveness of adding TMZ to radiotherapy in patients with methylated MGMT disease [12, 27]. To date, MGMT is the best known but not the only determinant in TMZ susceptibility.

The general understanding of DNA repairing mechanism, derived from DNA single-strand break or double-strand break, suggests activation of the other DNA damage repair (DDR) genes may also have roles, especially in lab research, but requires more evidences to elucidate [28]. As being the key factor to generate TMZ toxicity, the deficiency in MMR can have association with the resistance (Fig. 1). In a TCGA study, mutation of MSH6, a DNA mismatch repair gene, in post-treatment samples were identified to be associated with TMZ resistance [29]. In addition, this may have severe consequence by accelerating mutagenesis in resistant clones that could promote the neoplastic progression [30]. There was challenge in this theory, however, that only 3 % of 70 clinical samples showed single loci microsatellite instability (MSI) while no sample was with high MSI [31]. The rarity of alterations in promoter methylation was also confirmed by Felsberg, et al., showing only 9 in 80 patients and none in 43 patients had altered methylation in MGMT and in DNA mismatch repair genes including MLH1, MSH2, MSH6, and PMS2, respectively [32]. On the other hand, the authors found decreased expression of these MMR proteins in the recurrent tissue. Though controversy remains for each single factor to determine TMZ susceptibility, the DDRs associated with the modulation by TMZ in O^6^-MeG are suggested to have net impact in the resistance. As thus, recent studies put together MGMT, MMR, nucleotide excision repair (NER), homologous recombination (HR) for prediction of the drug response [33]. Finally, despite of fewer roles in N^7^-MeG to relate with TMZ drug cytotoxicity, cells proficient at repairing with base excision repair (BER) enzyme, the major targeting repairing protein, was also reported to cause resistance [2] (Fig. 1).

Setting up histological criteria based on the aforementioned DDRs is seemingly practicable and can contribute to personalized algorithm in guiding patients to receive the most appropriate treatment [23]. However, this only applies to prediction of drug response related to innate resistance, and is not avoidable for eventual treatment failure. In addition, the theory is only applicable of cellular level and ignores the impact of heterogeneity and the microenvironment that also compose for the tumor [34]. Regarding the complexity, it is doubtful that DDRs are fully responsible for all the mechanism in resistance. This is especially of noted in terms of acquired resistance, which the roles of DDRs remain ambiguous. Unlike the findings in rodent models [17], studies to date on whether anti-cancer therapies are responsible for inducing MGMT in humans have been inconclusive [35]. Much question remains regarding of cells to acquire resistance, suggesting more complicated and multi-factorial mechanisms.

## Dissecting the role of cancer stem-like cells in TMZ resistant mechanism

Limitation in DDR to fully explain the resistant mechanism gives rise to other theories such as cancer stem-like cells (CSCs) model, which represents specific subsets in the heterogeneous tumors accountable for acquiring treatment resistance and turning into more aggressive form [36]. These are characterized as the cells possessing self-renewal and multipotent properties [37]. The hypothesis suggests the cells possessing more intractable and resistant features, and is responsible for the disease recurrence after treatment [36]. In prostate cancer, for instance, enrichment of the CSC subpopulation after recurrence was characterized as lacking expression of androgen receptors and prostate specific antigen, indicating it to be no longer relying on hormone [38]. In GBM, this was supported by glioma cells carrying the stemness gene Nestin that could propagate the recurrence of GBM following TMZ treatment [39]. The specific subsets empowered with the features of stem-like cells were evidenced to take advantage in withstanding the treatment toxicity. The leukemia study showed that tumor specific treatment promotes outgrowth of the minor subsets with resistant power, with the initiating properties crucial in reserving the cells [40]. In a GBM study, tracing of the individual subpopulations showed their capability of phenotype adjustment, with outgrowth of specific ones determined by the adaptation speed to stress [41]. The theory remains worthwhile to define the phenomenon regarding explanation of the acquisition model in drug resistance.

Nevertheless, the theory often comes with controversy [42]. Inconsistent conclusion is often made between studies to studies because of the over-simplified methods for detection. For example, biomarker study without functional concerns may result in misleading recognition of the “CSC” subsets. Notably, application of CD133 as a universal phenotype marker in glioblastoma could be irrelevant [43]. This may be related to emergent understanding of the theory to have more commonality between clonal evolutions and CSC models [44], highlighting plasticity and dynamic of CSC features to adapt themselves against external microenvironment [45]. In other word, even the cells carrying the stemness features are also very dynamic and heterogenic in expression and function [46], and thus, application of single biomarker as representative of this specific group is insufficient [47]. In fact, in colon cancer, there were discordance between the stem cell-like features and the phenotype markers [48]. Regarding the complexity, comprehensive functional analysis to characterize the cells is hence mandatory [37], including functional characteristics such as substantial self-renewal, persistent proliferation, and tumor initiation. Specific methods, such as serial transplantation in a xenograft assay for self-renewal test, are applied to identify their ability [49]. Importantly, it is worth to note that cell lines often diminish or lose the ability of initiation and maintaining the cancer during long-term culture process. Even with keeping the stem-cell properties, the long-established cell lines were found to lose the nature of the disease such as incapable to form neuronal differentiation [50]. The relevant researches of these features thereby depends more on patient-derived xenograft tissue, which has the advantage in maintaining their properties because of avoiding differentiation by long-term culturing with serum-containing medium [50]. To summarize, functional studies of CSCs in cell lines can provide validation for stem-cell features [51].

Conventionally, drug resistance of CSCs is caused by abundant ATP-binding cassette (ABC) transporters expression in the cells. These proteins enable the cells to pump out drugs before intracellular damage occurs, allowing them to withstand the drug toxicity. The associations of the ABC transporters and CSCs are close, providing basic theory for “side population (SP) assay” to identify them [52]. This methodology somehow has limitation because certain normal tissue expresses the protein, and some CSC populations do not express it [53]. In regard with brain tissue, the ABC transporters is characterized between the barriers of blood and brain or blood and cerebrospinal fluid barrier, and is responsible for poor penetration of the drug [54, 55]. The fact turns to bring troublesome for applying SP assay to identify the CSC subpopulation in GBM since the barriers is often involved in the disease [56]. Of note, the specific cells isolated by SP assays in GBM cell lines and the primary tumor cells was not associated with cell capability of the self-renewal phenotype [57]. Further analysis of patient-derived xenograft revealed cells with SP identity was composed of the brain endothelial cells and was non-tumorigenesis [58]. These concluded the ABC transporters may not or are only partly involved in the resistant mechanism of GBM CSCs.

## Superior regulation of reactive oxygen species (ROS) in glioma stem**-**Like cells (GSCs)

Adaption of tumor cells is critical for their facing constantly fluctuating stress from internal environment or from anti-tumor treatment. With regard to TMZ, the drug itself and its analog TMZ-perillyl alcohol conjugate, were shown to up-regulate ROS production in GBM cells and non-small cell lung cancer cells, respectively [59, 60]. ROS is paradoxically critical both in the promotion of cancer progression and the induction of a detrimental cytotoxic response [61]. In fact, the main components of ROS include superoxide (O_2_^•−^), hydrogen peroxide (H_2_O_2_), and hydroxyl radical (^•^OH) [62]. Presence of superoxide is essential for cells to potentiate receptor tyrosine kinases (RTKs) such as EGFR and VEGFR [63, 64], in which the signaling aberrations are often related to the carcinogenesis of GBM [65]. As thus, several reports have shown that inhibition of ROS with N-acetylcysteine or ascorbic acid to decrease the risk of the disease [66–69]. On the contrary, unlike the physiology one, ROS generated by the treatment was through increase of excessive superoxide to induce the DNA damage response. Therefore, alleviation of ROS may mitigate the drug response, which interferes tumor control [70]. Multiple factors can be involved in this regulation. Nuclear factor-erythroid-2–related factor 2 (Nrf-2) is the most well-known regulator [71, 72]. In regard with TMZ, specific protein 1 (Sp1) was also triggered by ROS [73, 74], and was known to be a factor in the tolerance of the treatment-induced ROS [75]. This was through modulation of a ROS scavenging protein, superoxide dismutase (SOD) 2, and hence altered the regulation of oxidative stress and energy metabolism [76]. As thus, it is not surprising that certain cells possess an enhanced activity of antioxidant to tightly regulate ROS levels, thus maintaining viability and avoiding oxidative stress from anticancer therapy.

**Fig. 2:**
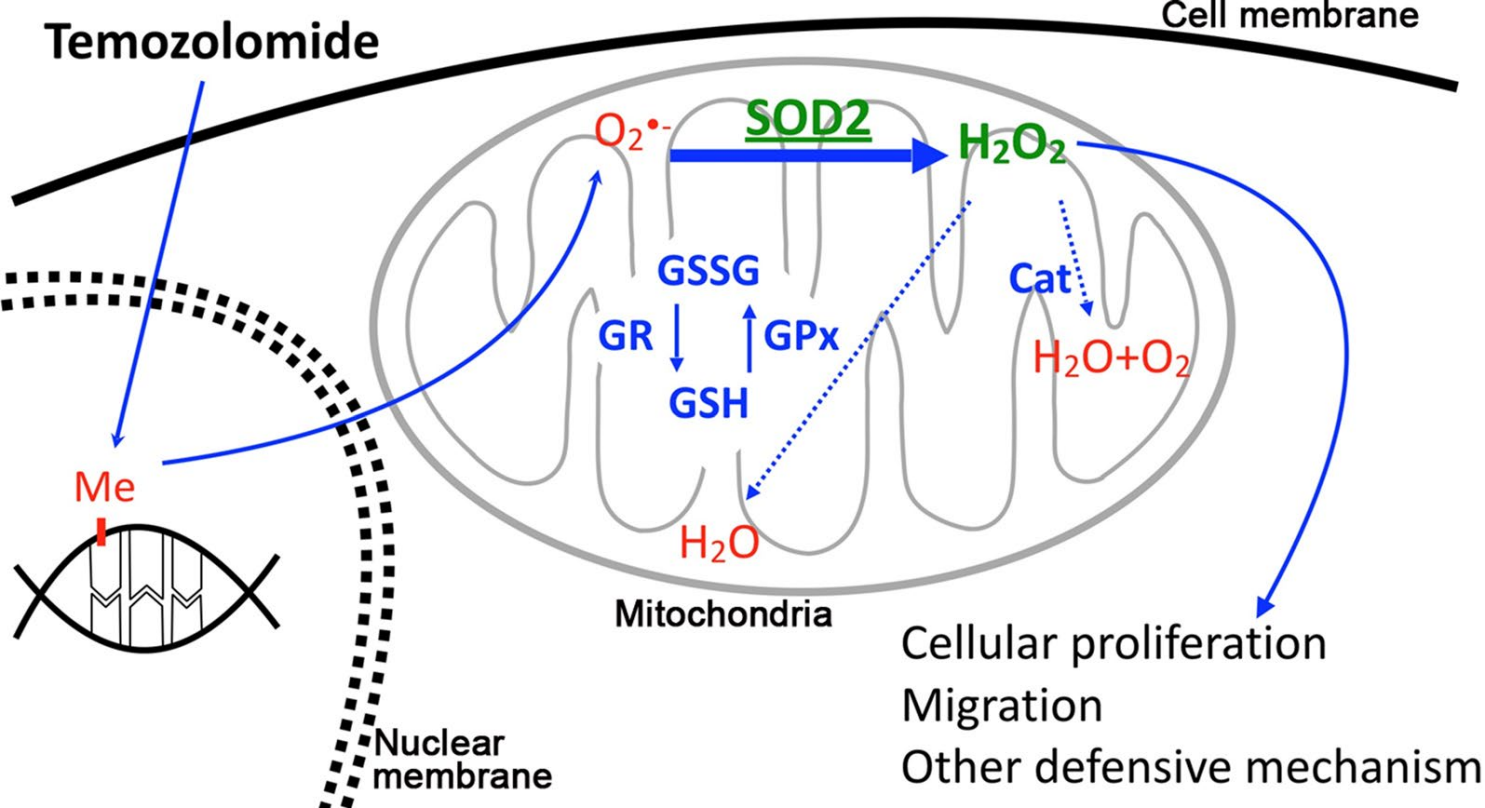
Schema of mechanism with involvement of ROS-related factors to take place in mitochondria in temozolomide (TMZ) resistance. Toxicity of TMZ induces excessive superoxide generation which requires activation of ROS scavengers to detoxify. Accumulation of ROS, on the other hand, promotes tumorigenesis. The two-handed sword effect of ROS induced by TMZ can end up in sparing the intractable cells that have superior regulatory ability. The remaining cells that survive chemotherapy will thus thaw and replace the vulnerable counterparts. *Cat* catalase, *GR* glutathione reductase, *GSH* glutathione, *GSSG* glutathione disulfide

Cell-based regulation of ROS is critical. Extrinsic sourced and endogenously generated reactive oxidants are continually infused to cells by balancing of metabolized oxygen. This is a physiologically regulated process, with deregulation leading to oxidative stress and pathologic consequence of cells [77]. Organelles such as mitochondria and endoplasmic reticulum, as well as enzymes including NADPH oxidases, xanthine oxidase, nitric oxide synthase and peroxisome, generate ROS. This leads to production of superoxide, which itself is only limited in impact. With the mid-product, secondary radical species can be generated after its variable reactions that yield multiple ROS and reactive nitrogen species, for example, hydrogen peroxide (H_2_O_2_) and peroxynitrite (ONOO^−^) [78]. Superoxide and its derivatives, especially H_2_O_2_, have crucial roles in promoting cellular proliferation, migration, and pathogen defensive mechanism [79] (Fig. 2). On the other hand, accumulation of the species can bring stress to cells. As thus, strict regulation of the product is essential to avoid oxidative damage from stress overload and for the maintenance of cell viability [80].

As of the regulatory mechanism, the ROS scavengers are the mainstay for the reaction. The protein family was composed of multiple enzymes including SODs, catalase, and glutathione peroxidase (GPx), with each having different role in the different stages required to convert superoxide into water and oxygen [81]. In regard with the first step of this reaction, three classes of dismutases are involved and distributed in different area of cells. SOD1 is spread in cytoplasm in majority to regulate the superoxide from NADPH oxidase reaction and cytochrome p450-monooxygenases in the endoplasmic reticulum. SOD2 is located in mitochondria, which the electron transport chain of mitochondria is the main sources to generate electron for superoxide formation. There is another dismutase, SOD3, located in the extracellular space, having various important roles in pathophysiology such as hypertension [82]. The proteins possess different catalytic metal ions, with SOD1 and SOD3 through Cu/Zn and SOD2 through Mn. By this reaction, superoxide is catalyzed to H_2_O_2_ and oxygen. The process continues with the hydrogen peroxide reduced to H_2_O and O_2_. This will need the involvement of catalase or GPx (Fig. 2).

Regarding the heterogeneity by cell-to-cell bases in tumor tissue, it is not surprising some cells have superior ability in regulating ROS. As aforementioned, the CSC subsets are with altered cellular features. Since external factors such as hypoxia plays crucial role in contribution of resistance in tumor tissue, the specific subsets can take advantage from the extreme condition [83]. GSCs, the CSCs specified in glioma, are preferably concentrated by perivascular, hypoxic, invasive niches regarding with the microenvironments [84]. The microenvironment was suggested to have role in treatment resistance, possibly through enrichment of GSCs [85, 86]. In neural stem cells, presence of hypoxia promotes cell differentiation and proliferation, suggesting how the external factor impacts [87]. In presence of hypoxia, the stemness features in GSC may lead to dominant expression of MGMT by increasing function of hypoxia inducible factors [88–90]. In addition, as being one of the stem cell factors, the polycomb group protein Bmi-1 was shown to have a crucial role in the GSCs homeostasis in response of ROS stress [91]. The activation of this factor is associated with radioresistance through the recruitment of DDRs [92]. Supportively, in a study by Garnier, et al., the appearance of DDRs was suggested to be a result of stochastic and unpredictable development from divergent evolution based on GSCs [93]. It is thus interesting that aberration of genetic repairing factors is related to CSCs and ROS in terms of resistance in GBM.

In cellular level, mitochondria are one of the most important organelles in adjusting against the external stress. It is not surprising for CSCs to have invigorated mitochondrial dynamics that offers advantage in withstanding the tough environment [94]. Mitochondria contribute to cancer in multiple aspects and seemingly have roles in progression and chemoresistance [95, 96]. Especially for treatment that works through generating excessive ROS such as TMZ treatment with or without radiotherapy, this would be of concern because the majority of ROS regulation in the cells takes place in mitochondria. In terms of the CSCs, the appearance of mitochondria differs from the differentiated cells by demonstrating underdeveloped structure such as more sparse and fragment with limited cristae. The CSCs also appears with less mitochondrial DNA. This is in reflex of cells to process high glycolytic flux and keeping itself in lower ROS, which may functionally support the self-renewal ability and the genome maintenance [94, 97]. With the acquisition of chemoresistance, a study showed that mitochondria responded to TMZ genotoxic stress with a major contribution from alternation of cytochrome c oxidase [98].

To maintain in a lowered ROS and possibly to withstand the cytotoxicity of cancer treatment, altered metabolic reprogramming by mitochondrial control in cancer may play a critical role [96, 99]. Many cancer cells rely on glycolysis over oxidative phosphorylation [100]. This is known as Warburg effect, which allows cancer cells to utilize the byproduct of glycolytic flux for synthesis of fatty acids, amino acids, and nucleotides. In addition, the production of lactate can be released to microenvironment and to feed adjacent oxidative cancer cells and to promote angiogenesis [101]. For the stemness-featured cells, glycolysis utilization is even more favored comparing to the differentiated cells [102]. Glycolysis reduces ROS production that can be destructive to DNA, keeping longevity of genome maintenance. More importantly, the derivations of cytosolic acetyl-CoA are critical for these cells to maintain histone acetylation and pluripotency [103]. With the feature, it is not surprising for CSCs, which possess superior regulation to maintain in lower ROS, to evade the effect of the treatment that delivers excessive ROS to the cells. Altogether, the theory highlights how the microenvironment and the anti-cancer therapy impact the CSC features to take advantage. In head and neck cancer cells, the specific subsets exhibited superior superoxide adjusting ability to withstand cisplatin treatment, which was also known to induce excessive ROS [104]. In GBM, we suggested that acquisition of TMZ resistance was a result of enriched cells with stem cell properties with enhanced SOD2 [105]. Finally, with the advanced methodology such as tracing system in the cells expressing stemness features, we expect the role of the specific subsets in drug resistance will be revealed in the future [106].

## Clinical implication regarding GSCs and TMZ resistance

As reviewed above, utilization of ROS and the altered mitochondria functioning in CSCs are critical for these specific subsets to survive and thrive from TMZ toxicity. This may also affect the effect of re-applying the current treatment or changing to the next-line treatment since many of the current therapy relied on ROS induction. For example, the ionizing radiation achieves tumor control by inducing oxidizing events that is harmful for DNA. Multiple chemotherapy agents achieve to control the disease by association with eliciting ROS reaction [107]. It was noted that induction of oxidative stress enhanced the effect of BCNU while presence of anti-oxidant alleviates the effect [71, 108]. Though not the standard reagent, the effect of widely-used cisplatin was also inferior in low-ROS exhibiting CSCs [104]. Recently approved TTFields contributes to the disease control by low-intensity electric fields that blocks cell division and interferes with organelle assembly [109]. The fact that sorafenib enhanced the effect of TTFields through increasing ROS to promote apoptosis also brings to worrisome in cross-resistance [110]. In general, replacement of the tumor cells that has superior capability in dealing with ROS can be troublesome in salvage treatment with the other anti-tumor strategies, which is a characteristic for the acquired resistance.

Strategies should be made to avoid the cross-resistance. Bevacizumab, which is an approved targeting therapy against VEGF, can be chosen with less impact in ROS. The other methods are active studying for tumor markers associated with anti-oxidant factors, such as Nrf-2 or SOD2. Direct modulation of ROS may also avoid the resistance or enhance the treatment [111]. Down-regulation of glutathione reductase can resensitize the resistant GBM cells to TMZ or cisplatin [112]. In our previous studies, application of Sp1 or SOD inhibitor could down-regulate TMZ-induced ROS and promoted the cytotoxicity of resistant cells [75]. Metabolism of glycolysis is served as new target [113]. In addition, recent understanding of autophagy in protecting the cells from cytotoxicity also reveals the association with ROS and mitochondrial alteration [114–117]. As thus, application of chloroquine treatment to block autophagy can induce the increased production of intracellular or mitochondrial ROS [118, 119]. This will also cause accumulation of damaged mitochondria or oxidative stress [120, 121]. Finally, the plasticity model for CSC suggests epigenomic regulation crucial for the features to convert in cells [122]. With the enthusiasm of CSC studies recently, targeting the relative factors is on the way for clinical trials [123]. As thus, specific strategies such as applying a histone deacetylases inhibitor, suberoylanilide hydroxamic acid, could be studied for the potential strategies in reducing or overcoming the resistance [124].

## Conclusions

Limited breakthrough has been achieved in GBM treatment in the past few years. Before the major leap to happen, inspection of the tumor biology is potentially contributable in optimizing treatment to improve the disease outcome and in developing novel therapeutic strategies. Regarding the underlying resistant mechanism of TMZ, rationales were suggested to combine the first-line drug along with simultaneous suppression over the higher protection mechanisms of the stem-like cells to seek for opportunity to enhance the drug efficacy. This can possibly be achieved by modulating selective factors related to these specific subsets, such as enhancement of antioxidant enzymes, energy metabolism, and adaptation in the microenvironment. Though the inhibition may not be straightforward, the rationale suggests a potential framework to benefit cancer treatment.

Abbreviations.

GBM: glioblastoma multiforme; TMZ: temozolomide; BCNU: carmustine; TTFields: tumor-treating fields device; IDH: isocitrate dehydrogenase; Nrf-2: nuclear factor-erythroid-2–related factor 2; Sp1: specific protein 1; SOD2: superoxide dismutase 2; ROS: reactive oxygen species; H_2_O_2_: hydrogen peroxide; GPx: glutathione peroxidase, MGMT: O^6^-methylguanine-DNA methyltransferase; TCGA: The Cancer Genome Atlas; MSI: microsatellite instability; CSC: cancer stem-like cell; GSC: glioma stem-like cell; SP: side population; ABC: ATP-binding cassette; DDR: DNA damage repair; NER: nucleotide excision repair; HR: homologous recombination; BER: base excision repair; RTK: receptor tyrosine kinase; EGFR: epidermal growth factor receptor; VEGFR: vascular endothelial growth factor receptor.

## Data Availability

Not applicable.
